# Anemia and Its Associated Factors Among Children Aged 6–59 Months After Internal Displacement in Central Sudan: A Cross-Sectional Study

**DOI:** 10.3390/healthcare14193217

**Published:** 2026-09-29

**Authors:** Hatim G. Altahir, Mohammad Alhasoon, Ebtihal E. Eltyeb, Abdullah Al-Nafeesah, Ashwaq AlEed, Ishag Adam

**Affiliations:** 1 Department of Pediatrics, Singa Hospital, Singa 25511, Sudan; hatimgindeel6@gmail.com; 2 Department of Pediatrics, College of Medicine, Qassim University, Buraydah 52571, Saudi Arabia; m.alhasoon@qu.edu.sa (M.A.);; 3 Faculty of Medicine, Jazan University, Jazan 45142, Saudi Arabia; eeltyeb@jazanu.edu.sa; 4 Department of Obstetrics and Gynecology, College of Medicine, Qassim University, Buraydah 52571, Saudi Arabia; ia.ahmed@qu.edu.sa

**Keywords:** anemia, internal displacement, childhood anemia, children under five, Sudan conflict, maternal age

## Abstract

**Background:** The ongoing conflict in Sudan has caused internal displacement. While pre-war baselines have been established, a critical gap remains in the literature on pediatric health in post-conflict reclaimed urban centers. This study aimed to determine the prevalence of anemia and identify its associated factors among children aged 6–59 months in Singa, Central Sudan. **Methods:** A community-based, multistage stratified cross-sectional study was conducted in Singa, Central Sudan, between August and September 2025. Sociodemographic characteristics were collected through face-to-face interviews. Anemia was defined according to the 2024 WHO age-specific hemoglobin criteria. Multivariable binary logistic regression analysis was performed. **Results:** A total of 450 children aged 6–59 months were included in the study. The median age was 23.5 months (interquartile range [IQR]: 13.0–38.0), and 252 (56.0%) were male. Most children were delivered vaginally (65.6%), had mothers with at least secondary education (53.1%), and were completely immunized (76.7%). The prevalence of anemia was 74.4% (95% confidence interval [CI] = 70.4–78.4%). In the univariable binary logistic regression analysis, younger age, smaller family size, younger maternal age, lower body weight, and a recent history of diarrhea were associated with anemia. After adjustment for potential confounders, while maternal age was inversely associated with anemia (adjusted odds ratio (aOR) = 0.95, 95% CI: 0.92–0.99) a recent history of diarrhea (aOR = 1.97, 95% CI: 1.22–3.17) was positively associated with anemia. The multivariable model demonstrated good calibration (Hosmer–Lemeshow *p* = 0.177). **Conclusions:** Anemia is a major public health concern among children aged 6–59 months returning to post-displacement settings. Anemia is primarily driven by younger maternal age and recent acute infectious diarrhea. Integrated strategies focusing on maternal education and support, diarrhea prevention and management, and early identification of at-risk children are needed to reduce the burden of anemia in this population.

## 1. Introduction

Anemia is a major global public health problem affecting millions of children worldwide [1]. It is characterized by a significant reduction in hemoglobin concentration or a deficit in the total volume of circulating red blood cells, which can adversely affect cognitive function, physical growth, and overall child development [1]. Anemia was defined according to the 2024 World Health Organization guideline on hemoglobin cutoffs as a hemoglobin concentration <10.5 g/dL for children aged 6–23 months and <11.0 g/dL for children aged 24–59 months [2].

During early childhood, a critical window of rapid neurodevelopmental trajectory and physiological maturation, the consequences of hematological deficiency are particularly severe and long-lasting [3]. Prolonged anemia during this vital developmental phase has been definitively linked to irreversible delays in cognitive function, poor fine and gross motor skills, impaired behavioral development, and an elevated baseline susceptibility to severe childhood infections [3]. Consequently, it acts as a silent but potent driver of childhood under-five morbidity and mortality, disproportionately burdening populations trapped within cycles of intergenerational poverty, structural inequality, and systemic food insecurity across low- and middle-income countries [4]. Across East and North Africa, the burden of childhood anemia is exceptionally high, sustained by an overlapping matrix of macro-structural problems, including unstable localized food distribution networks, environmental shifts, and fragile public healthcare systems [3,5,6]. Epidemiological evidence from across sub-Saharan Africa (SSA) reveals a shared vulnerability among pediatric populations exposed to acute humanitarian crises and baseline developmental deficits [3,5]. Studies in neighboring nations such as Ethiopia [7,8] and Tanzania [9] consistently underscore the complex, multi-factorial etiology of anemia. Furthermore, macro-level evidence from SSA, including Sudan, indicates that severe state-level gaps in under-five mortality are driven by structural social determinants of health, such as maternal literacy, household income, and access to improved water, sanitation, and hygiene (WASH) [10,11]. SSA bears an exceptionally high burden of pediatric hematological deficiency; the pooled regional prevalence of anemia among children aged 6 to 59 months is estimated at 64.1% (95% CI: 63.9%–64.4%) [3]. This substantial burden is reflected in wide sub-continental variations, ranging from 35.3% in southern Ethiopia [7] and 39.0% in Central Ethiopia [8], to 49.4% in Sudan [12], and peaking at 67.4% in Uganda [13]. This epidemiological profile is shaped by a dense network of sociodemographic, dietary, and biological risk factors. Specifically, factors such as child age [6,7,11], child sex [3,9], having diarrhea in the last two weeks [3,14], low maternal educational level, and larger household sizes [3] have been reported to be associated with anemia. This delicate physiological equilibrium is completely upended in zones of active warfare and civil unrest, where forced internal displacement acts as a powerful catalyst for acute medical emergencies [15]. The ongoing conflict in Sudan, which erupted in April 2023, has rapidly transformed into one of the most severe humanitarian disasters in modern history [16]. The conflict has displaced people and severely compromised the health system, disrupting primary healthcare services, disease control programs, water treatment infrastructure, and routine child health interventions [6]. Sennar State, and specifically the strategic town of Singa, has been an active theater of this conflict, enduring shifts in military control and widespread destruction before being reclaimed [17]. Returning populations find themselves in a profoundly compromised environment, forced to rebuild livelihoods from scratch while navigating severe shortages of clean drinking water, sanitation facilities, clothing, and micronutrient-dense foods. Furthermore, conflicts systematically drive infectious disease emergence through infrastructure destruction and displaced populations [18,19], directly fueling central vulnerabilities like acute waterborne diarrheal outbreaks among returned children. Moreover, multi-country evidence shows that inadequate sanitation and unimproved water access increase overall under-five mortality across SSA [20], mirroring the immediate localized threats facing returned children in Singa. Under post-conflict emergency conditions, the biological pathways driving hematological deficiency are often accelerated [19,21].

While historical data describe the baseline patterns of nutritional deficiencies and childhood anemia during periods of relative stability in Sudan [12,22], village-level data from Northern Sudan also documented a similarly high prevalence of child anemia, at 80.4% [22], confirming localized baseline vulnerabilities. However, there is a critical gap in the peer-reviewed literature regarding the post-conflict, post-displacement reality. There is virtually no current data evaluating the exact prevalence and predictors of anemia among under-five children who have returned to highly compromised urban centers like Singa following internal displacement. Given the high volatility of these environments, relying on pre-war epidemiological baselines can result in poorly targeted humanitarian responses. Accurate, timely field data are essential for understanding the modern risk factors facing these children and for allocating scarce medical and nutritional resources effectively.

To address this information gap, this community-based study was conducted to determine the prevalence of anemia and its independent sociodemographic and clinical risk factors among children under five years of age in Singa, Central Sudan, during the post-displacement period of late 2025. Filling such gaps is essential to provide local health authorities, non-governmental organizations, and international aid agencies with the empirical foundation required to implement targeted nutritional support and clinical protocols tailored to this highly vulnerable population.

## 2. Materials and Methods

This community-based cross-sectional study was conducted in Singa, Sudan. Singa is a town situated in the Sennar State, Sudan, at an elevation of 439 m above sea level. The town is situated on the west bank of the Blue Nile, 360 km southeast of the capital, Khartoum, and 60 km from the city of Sennar. It has a population of 92,450 in 2026 [23]. There are seven administrative units in Singa [23]. On 29 June 2024, the city was captured by the Rapid Support Forces during the ongoing Sudanese war, and in November 2024, the Sudanese Armed Forces retook the city [24]. After returning, internally displaced people had to rebuild their lives from scratch and share their remaining resources, subsisting on minimal amounts of food, water, and clothing [24].

### 2.1. Study Population

This multistage community-based cross-sectional survey was conducted from August to September 2025 according to Strengthening the Reporting of Observational Studies in Epidemiology [25] (Supplementary File S1). Using a stratified sampling technique, four of the seven administrative sectors were selected by simple random sampling (lottery method). Households within each selected administrative sector were then randomly selected using the lottery method. The number of selected households was in proportion to their population sizes. One eligible child aged 6–59 months was randomly selected from each household. If a selected household had no eligible child or the parent/guardian declined participation, the next household was approached. Although the research team did not provide direct medical care, children identified with severe anemia or other acute medical conditions during data collection were referred to the nearest appropriate healthcare facility for further evaluation and management.

### 2.2. Inclusion and Exclusion Criteria

The participants were children aged 6–59 months, including both males and females, whose parents or guardians provided informed consent for their participation in this study. Children with severe congenital anomalies, chronic conditions known to affect hemoglobin status (e.g., chronic kidney disease or malignancy), or those receiving cytotoxic chemotherapy or immunosuppressive therapy were excluded. Children receiving routine treatment for anemia, including iron supplementation, remained eligible for inclusion.

### 2.3. Data Collection

The data were collected by trained medical doctors (two males and two females) using a structured questionnaire administered in Arabic (the official language in the setting) during face-to-face interviews. The instrument captured the child’s age (extracted from the date of birth and confirmed with the birth certificate), sex, maternal age, parental education level and occupation, mode of delivery, birth spacing, family size, duration of breastfeeding, history of diarrhea in the preceding two weeks, and complete childhood immunization status (as per age).

### 2.4. Anthropometric and Hemoglobin Measurements

Using standard procedures, the children’s weights and heights were measured twice, and the average of the two measurements was recorded. Weight was measured in kilograms using a Seca 874 weighing scale (Seca, Hamburg, Germany) to the nearest 100 g. The scale was calibrated and zero-adjusted before each measurement, with shoes and heavy outer clothing removed. Height was measured to the nearest 0.1 cm using a Seca stadiometer (Shorr Board, Chino, CA, USA) for children who could stand upright independently. For infants and children under two years of age, length was measured in a recumbent position using a standard infant measuring board calibrated in centimeters. The WHO reference was used to compute the body mass index (BMI) z-score using variables for stunting (low height for age) and the BMI z-score [26,27].

Hemoglobin concentration was measured from capillary blood samples obtained via a finger prick or heel stick using an aseptic technique. Measurement was carried out on-site using a portable, pre-calibrated HemoCue photometer (HemoCue, Ängelholm, Sweden). Anemia was defined according to the 2024 World Health Organization (WHO) age-specific hemoglobin thresholds: hemoglobin <10.5 g/dL for children aged 6–23 months and <11.0 g/dL for those aged 24–59 months [2]. Based on these age-specific cutoffs, participants were classified into two categories: anemia and no anemia. Anemia status served as the primary outcome variable in subsequent analyses.

### 2.5. Sample Size

A sample size of 450 children was calculated using the OpenEpi software v.3.01 menu (OpenEpi, Atlanta, GA, USA) [28]. The calculation was based on the single-population proportion formula:

n_0_ = (Z^2^ × p × (1 − p))/d^2^

where n_0_ is the required sample size for a simple random sample, Zα/2 = 1.96 corresponds to a 95% confidence level, *p* = 0.50 is the assumed prevalence of anemia, and d = 0.05 is the desired margin of error. An expected prevalence of 50% was conservatively selected because it yields the maximum required sample size, and this was assumed based on previous reports on anemia in under-five children in Africa [3]. Depending on the previous report, we further assumed that 52.0% of children with anemia and 40.0% of children without anemia would have a history of diarrhea in the past two weeks, respectively.

### 2.6. Statistical Analysis

Data were analyzed using SPSS (v. 25; IBM Corp., Armonk, NY, USA). Continuous data, including child’s age, birth spacing, breastfeeding duration, maternal age, family size, weight, and height, were assessed for normality using the Shapiro–Wilk test. Because the continuous variables exhibited non-normal distributions, they were reported as medians with interquartile ranges (IQRs). Categorical variables and the overall prevalence of anemia were expressed as frequencies and percentages.

Univariable binary logistic regression analysis was performed with anemia status (yes/no) as the dependent variable. The independent sociodemographic and clinical variables included child’s age, sex, birth spacing, breastfeeding duration, maternal age, family size, weight, height, BMI z-score, maternal/paternal education levels, maternal occupation, mode of delivery, history of diarrhea in the past two weeks, and immunization status. We decided to include the child’s age, sex, maternal age, family size, and history of diarrhea in the past two weeks in the multivariable binary regression analysis regardless of their *p*-values in the univariable binary analysis. Thereafter, variables with a *p*-value < 0.20 in the univariable analysis were entered into a multivariable binary logistic regression model to identify the associated factors for anemia. A total of six candidate variables (child’s age, sex, family size, maternal age, weight, and recent history of diarrhea) were simultaneously entered into the final model using the Enter method. The study included 335 anemia events, corresponding to approximately 56 events per variable, exceeding the recommended minimum of 10 events per variable for logistic regression. Multicollinearity among the independent variables was assessed (variance inflation factor > 4) before model fitting and was not detected. Model performance was evaluated using the Omnibus test of model coefficients and the Hosmer–Lemeshow goodness-of-fit test. Adjusted odds ratios (aORs) with 95% confidence intervals were reported, and a two-sided *p*-value < 0.05 was considered statistically significant.

## 3. Results

**Figure 1:**
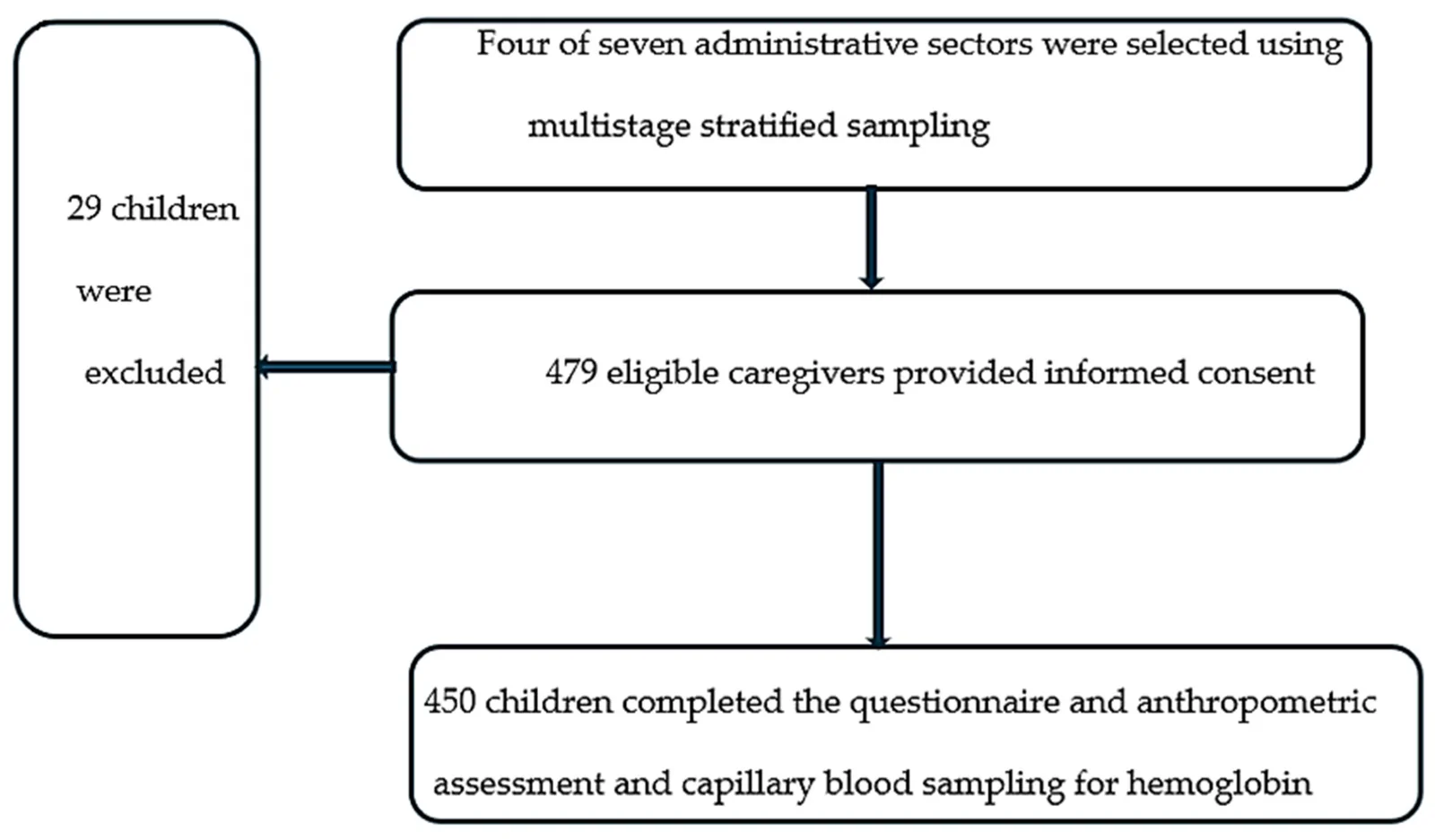
Flow diagram illustrating participant recruitment and selection in this community-based multistage stratified cross-sectional study conducted in Singa, Central Sudan.

A total of 479 children were initially assessed for eligibility. During data cleaning, 29 records were excluded due to ineligible ages (outside the predefined range of 6–59 months), missing age information, or implausible hemoglobin values attributable to data entry errors. The final analytical sample therefore comprised 450 children, as shown in Figure 1.

A total of 450 children under five years of age had complete data and were included in the analysis. The median age of the participants was 23.5 months (IQR: 13.0–38.0). Of the study population, 225 (50%) were aged 6–23 months and 225 (50%) were aged 24–59 months. The median birth spacing was 24.0 months (IQR: 12.0–36.0), and the median duration of breastfeeding was 18.0 months (IQR: 12.0–22.0). The median maternal age was 28.0 years (IQR: 24.0–33.0), and the median family size was 5.0 members (IQR: 4.0–6.0). The median weight and height of the children were 9.95 kg (IQR: 7.8–12.5) and 80.0 cm (IQR: 72.0–92.0), respectively.

Regarding sociodemographic characteristics, 56.0% of the children were male. The majority were delivered vaginally (65.6%). More than half of the mothers (53.1%) had at least secondary education, and most were housewives (86.2%). Similarly, 54.9% of fathers had at least a secondary education. A history of diarrhea in the previous two weeks was reported by 68.7% of the children, and 76.7% were fully immunized.

Of the 450 children included in the analysis, 335 (74.4%, 95% CI = 70.4–78.4%) were classified as having anemia according to the 2024 WHO age-specific hemoglobin thresholds, whereas 115 (25.6%) had normal hemoglobin levels. (Table 1).

Table 2 presents the univariable binary logistic regression analysis of factors associated with anemia among children under five years of age. Children with anemia were significantly younger than those without anemia, with a median age of 22.0 months (IQR: 13.0–36.0) compared with 29.0 months (IQR: 15.0–46.0) (OR = 0.98, 95% CI: 0.97–0.99). Family size was inversely associated with anemia (OR = 0.89, 95% CI: 0.78–0.99). Similarly, increasing maternal age was inversely associated with anemia (OR = 0.94, 95% CI: 0.91–0.97). Children with anemia also had a lower median body weight than those without anemia, and increasing weight was associated with reduced odds of anemia (OR = 0.93, 95% CI: 0.86–0.99). Children with a recent history of diarrhea had more than twice the odds of anemia compared with those without diarrhea (OR = 2.28, 95% CI: 1.47–3.54). In contrast, birth spacing, breastfeeding duration, height, sex, maternal education, maternal occupation, paternal education, mode of delivery, and immunization status were not associated with anemia (*p* > 0.05).

In the multivariable binary logistic regression analysis, maternal age was inversely associated, and recent history of diarrhea was positively associated with anemia (Table 3). Each 1-year increase in maternal age was associated with a 4.8% reduction in the odds of anemia (aOR = 0.95, 95% CI: 0.92–0.99). Children with a recent history of diarrhea had approximately twofold higher odds of anemia than those without diarrhea (aOR = 1.97, 95% CI: 1.22–3.17). Child age, family size, weight, and sex were not associated with anemia after adjustment.

## 4. Discussion

This community-based study offers a critical field assessment of the prevalence and predictors of anemia among children under five years of age returning to Singa, Central Sudan, following severe conflict-driven internal displacement. Our findings reveal a high prevalence of anemia, at 74.4%, among the sampled pediatric population. This figure dramatically surpasses the pre-war Sudanese national baseline of 49.4% reported by Elmardi et al. [12] and significantly exceeds burdens observed in stable, non-conflict settings within neighboring East African countries, such as Central Ethiopia (39.0%) [8] and Southern Ethiopia (35.3%) [7].

Instead, the anemia documented in post-displacement Singa aligns closely with observations from active conflict zones and internally displaced persons’ complexes, such as the Debark refugee camp in Northwest Ethiopia, where pediatric anemia reached 50.3% under acute structural threat [15]. Most notably, this finding closely mirrors historical rural village-level data from the Northern State of Sudan, which recorded a localized child anemia prevalence of 80.4% [22]. Taken together, these comparisons indicate that while macro-level social determinants historically establish structural vulnerabilities across Sudan [10,11], the severe infrastructure collapse and resource scarcity following the 2023 war have effectively normalized these extreme, worst-case epidemiological baselines in returned urban centers like Singa.

Multivariable binary logistic regression revealed maternal age was inversely associated with anemia (AOR = 0.95, 95% CI: 0.92–0.99). The observed inverse association between maternal age and anemia is consistent with evidence from a previous study in sub-Saharan Africa, suggesting that children of younger mothers may be at greater risk of anemia [3,12]. Although the underlying mechanisms were not examined in the present study, younger maternal age may reflect differences in caregiving experience, childcare practices, health literacy, or socioeconomic circumstances that could indirectly influence child health and nutritional outcomes. Younger mothers may also encounter greater challenges in accessing healthcare services and implementing optimal infant and young child feeding practices, particularly in resource-constrained settings. However, these potential explanations should be interpreted cautiously, as the present study did not collect detailed information on maternal nutritional knowledge, childcare behaviors, household food security, or healthcare utilization. Therefore, the observed association should be considered epidemiological rather than causal, and further prospective studies are needed to clarify the pathways linking maternal age and childhood anemia.

Our final model also identified that a history of acute diarrhea within the preceding two weeks was associated with anemia (AOR = 1.97, 95% CI: 1.22–3.17). The prevalence of diarrhea in this study (68.7%) was higher than previously reported in Ethiopia [7]. The destruction of local water treatment networks and sanitation systems in Singa forces returning households to subsist on unimproved water facilities or surface water from the Blue Nile, conditions known to drastically elevate under-five mortality across the subcontinent [20].

Pathogenesis from frequent bouts of acute diarrhea triggers massive malabsorption of essential dietary micronutrients such as iron, vitamin B12, and folate [29]. Concurrently, disruptions in standard childhood vaccination campaigns leave children exposed to preventable infections, further straining their nutritional and hematological reserves [30]. Biologically, these frequent diarrheal bouts explain the high rates of anemia via nutrient malabsorption [29] and hepcidin-mediated iron sequestration [31,32], which effectively starves the bone marrow during critical growth phases. This severe pathway closely matches regional historical trends in Eastern Sudan, where gastroenteritis stands as the primary cause of childhood medical hospitalization [33], emphasizing that unmitigated waterborne illness consistently drives severe systemic pediatric morbidity in this region.

Several sociodemographic variables such as maternal and paternal education levels, family size, and maternal occupation lost their statistical significance after multivariable adjustment, despite their association in previous studies [3,6,11]. Wartime displacement caused universal socio-economic destruction, overriding the protective benefits of parental literacy and employment. These shared environmental challenges may have diminished socioeconomic differences in exposure to risk factors for anemia, which could partly explain the lack of significant associations between several socioeconomic variables and childhood anemia observed in this study.

### Strengths and Limitations

This study has several strengths. It provides community-based data on the burden of anemia among children under five years of age in a post-displacement setting in Central Sudan, a population for which contemporary epidemiological data are scarce. The multistage stratified sampling approach enhanced the representativeness of the study population. The hemoglobin concentration was measured using a standardized point-of-care HemoCue photometer, providing reliable field-based assessment of anemia. Several limitations should also be acknowledged. First, the cross-sectional design precludes establishing temporal or causal relationships between the identified factors and anemia. Second, anemia was defined solely by age-specific hemoglobin concentrations, in accordance with the 2024 World Health Organization guidelines. Additional laboratory investigations, such as serum ferritin, inflammatory markers, red blood cell indices, or other hematological tests, were not performed; therefore, the underlying etiology of anemia could not be determined, and iron deficiency could not be distinguished from other causes. Third, several important determinants of childhood anemia, including dietary quality, intake of iron-rich foods, iron supplementation, parasitic infections, household food security, and access to healthcare, were not assessed and therefore could not be included in the multivariable analysis, leaving the possibility of residual confounding. Fourth, breastfeeding duration and the recent history of diarrhea were based on caregiver recall and may be subject to recall bias. Although the multivariable model adjusted for several demographic and clinical factors, residual confounding from unmeasured variables cannot be excluded.

The study very strongly links the observed anemia to the period following displacement. However, there is no control group or pre-displacement measurement for the same children or population. We compared the figure of 74.4% with the historical prevalence of 49.4% for Sudan and, on this basis, suggest an impact of the conflict. Although such a comparison is interesting from a descriptive standpoint, it cannot serve as a basis for causal inferences, as the populations, time periods, regions, and potentially measurement methods differ. Future prospective studies incorporating comprehensive nutritional assessment, standardized anthropometric indices, laboratory investigations to determine the etiology of anemia, and longitudinal follow-up are needed to better characterize the determinants of childhood anemia and inform targeted prevention and management strategies in post-conflict settings.

## 5. Conclusions

In conclusion, anemia is a major public health problem among children under five years of age in Singa, Central Sudan, affecting nearly three-quarters of the study population. After adjustment for potential confounders, younger maternal age and a recent history of diarrhea were associated with anemia. These findings underscore the importance of strengthening maternal and child health services, improving the prevention and management of diarrheal diseases, and implementing targeted anemia screening and nutritional interventions for young children. Comprehensive public health strategies integrating nutrition, infection prevention, and early detection are needed to reduce the burden of childhood anemia in this resource-limited setting.

## Figures and Tables

**Table 1:** General Characteristics of the Studied Children in Singa, Central Sudan.

Variables		Median	Interquartile Range
Age, months		23.5	13.0–38.0
Birth spacing, months		24.0	12.0–36.0
Breastfeeding, months		18.0	12.0–22.0
Mother’s age, years		28.0	24.0–33.0
Family size		5.0	4.0–6.0
Weight, Kilogram		9.95	7.8–12.5
Height, Centimeter		80.0	72.0–92.0
Body mass index-Z-score		−0.84	−2.02–0.05
		**Frequency**	**Percentage**
Sex	Male	252	56.0
	Female	198	44.0
Mode of delivery	Vaginal	295	65.6
	Cesarean	155	34.4
Mother education	≥secondary level	239	53.1
	˂secondary level	211	46.9
Mother occupation	Housewife	388	86.2
	Working	62	13.8
Father education	≥secondary level	247	54.9
	˂secondary level	203	45.1
History of diarrhea in the last two weeks	Yes	309	68.7
	No	141	31.3
Complete immunization	Yes	345	76.7
	No	105	23.3
Anemia	Yes	335	74.4
	No	115	25.6

**Table 2:** Univariable binary logistic regression analysis of Factors Associated with Anemia Among Children Under Five Years of Age in Singa, Central Sudan.

Variables		Children with Anemia (Number = 335)	Children Without Anemia (Number = 115)	Odds Ratio	95% Confidence Interval	*p* Value
	Median (Interquartile Range) of					
Age, months		22.0 (13.0–36.0)	29.0 (15.0–46.0)	0.98	0.97–0.99	0.008
Birth spacing, months		24.0 (12.0–36.0)	24.0 (9.0–36.0)	1.00	0.99–1.01	0.348
Family size		5.0 (4.0–6.0)	5.0 (4.0–7.0)	0.89	0.78–0.99	0.029
Mother’s age, years		27.0 (23.0–32.0)	30.0 (25.0–35.0)	0.94	0.91–0.97	<0.001
Breastfeeding, months		18.0 (12.0–23.0)	18.0 (14.0–22.0)	0.99	0.96–1.03	0.791
Height, Centimeter		79.5 (72.0–91.0)	86.0 (72.0–98.0)	1.00	0.99–1.00	0.658
Weight, Kilogram		9.8 (7.6–12.2)	10.5 (8.1–13.1)	0.93	0.86–0.99	0.029
Body mass index-Z-score		−1.12 (−2.15–0.18)	−0.68 (−1.81–−0.30)	0.99	0.92–1.09	0.924
Sex	Male	194 (57.9)	58 (50.4)	Reference	0.48–1.13	0.164
	Female	141 (42.1)	57 (49.6)	0.74		
Mother’s education	≥secondary level	176 (52.5)	63 (54.8)	Reference	0.72–1.68	0.677
	˂secondary level	159 (47.5)	52 (45.2)	1.10		
Mother’s occupation	Housewife	289 (86.3)	99 (86.1)	Reference	0.53–1.82	0.961
	working	46 (13.7)	16 (13.9)	0.99		
Father’s education	≥secondary level	188 (56.1)	59 (51.3)	Reference	0.54–1.26	0.371
	˂secondary level	147 (43.9)	56 (48.7)	0.82		
Mode of delivery	Vaginal	215 (64.2)	80 (69.6)	Reference	0.81–2.01	0.295
	Cesarean	120 (35.8)	35 (30.4)	1.28		
History of diarrhea in the last two weeks	Yes	246 (73.4)	63 (54.8)	Reference	1.47–3.54	<0.001
	No	89 (26.6)	52 (45.2)	2.28		
Complete immunization	Yes	254 (75.8)	91 (79.1)	Reference	0.70–1.94	0.555
	No	81 (24.2)	24 (20.9)	1.17		

**Table 3:** Multivariable Binary Logistic Regression Analysis of the Factors Associated with Anemia Among Children in Singa, Central Sudan.

Variable	Adjusted Odds Ratio	95% Confidence Interval	*p* Value
Age (months)	0.99	0.97–1.02	0.507
Male sex	1.39	0.89–2.17	0.144
History of diarrhea (Yes vs. No)	1.97	1.22–3.17	0.005
Family size	0.98	0.86–1.11	0.734
Maternal age (years)	0.95	0.92–0.99	0.013
The child’s weight (kg)	1.02	0.89–1.16	0.803

## Data Availability

The data presented in this study is available on request from the corresponding author. The data is not publicly available due to privacy and ethical restrictions.

## Supplementary Materials

The following supporting information can be downloaded at: https://www.mdpi.com/article/10.3390/healthcare14193217/s1, File S1: STROBE Statement—checklist of items that should be included in reports of observational studies.
