# Supplementary material for: Anemia and Its Associated Factors Among Children Aged 6–59 Months After Internal Displacement in Central Sudan: A Cross-Sectional Study
Source: Healthcare (Basel). 2026 Sep 29;14(19):3217. doi: 10.3390/healthcare14193217 (PMC13665207; doi:10.3390/healthcare14193217)
Supplement: Supplementary file 1 [file healthcare-14-03217-s001.zip › healthcare-4519888-supplementary.pdf]

STROBE Statement—checklist of items that should be included in reports of observational studies

	Item No.	Recommendation	Page No.	Relevant text from manuscript
**Title and abstract**	1	(*a*) Indicate the study's design with a commonly used term in the title or the abstract	Title; Abstract 1	Study design identified in the title and abstract; abstract provides a balanced summary.
		(*b*) Provide in the abstract an informative and balanced summary of what was done and what was found	Title; Abstract 1	Study design identified in the title and abstract; abstract provides a balanced summary.
**Introduction**				
Background/rationale	2	Explain the scientific background and rationale for the investigation being reported	Introduction 1	Scientific background and rationale described.
Objectives	3	State specific objectives, including any prespecified hypotheses	Introduction	Study objectives clearly stated.
**Methods**				
Study design	4	Present key elements of study design early in the paper	Methods 3	Community-based cross-sectional study design described.
Setting	5	Describe the setting, locations, and relevant dates, including periods of recruitment, exposure, follow-up, and data collection	Methods 3	Setting, location, and study period reported.
Participants	6	(*a*) *Cohort study*—Give the eligibility criteria, and the sources and methods of selection of participants. Describe methods of follow-up<br>*Case-control study*—Give the eligibility criteria, and the sources and methods of case ascertainment and control selection. Give the rationale for the choice of cases and controls<br>*Cross-sectional study*—Give the eligibility criteria, and the sources and methods of selection of participants	Methods 3	Eligibility criteria and participant selection described.
		(*b*) *Cohort study*—For matched studies, give matching criteria and number of exposed and unexposed<br>*Case-control study*—For matched studies, give matching criteria and the number of controls per case	Methods 3,4	Eligibility criteria and participant selection described.

Variables	7	Clearly define all outcomes, exposures, predictors, potential confounders, and effect modifiers. Give diagnostic criteria, if applicable	Methods 4	Outcome, exposures, predictors, and diagnostic criteria defined.
Data sources/ measurement	8*	For each variable of interest, give sources of data and details of methods of assessment (measurement). Describe comparability of assessment methods if there is more than one group	Methods 4	Measurement methods including HemoCue hemoglobin assessment described.
Bias	9	Describe any efforts to address potential sources of bias	Methods 4	Potential sources of bias addressed.
Study size	10	Explain how the study size was arrived at	Methods 4	Sample size calculation and design effect described.

Quantitative variables	11	Explain how quantitative variables were handled in the analyses. If applicable, describe which groupings were chosen and why	Methods 4	Handling of quantitative variables and age categories described.
Statistical methods	12	(*a*) Describe all statistical methods, including those used to control for confounding	Methods 4	Statistical analyses, multivariable logistic regression, confounding control, and diagnostics described.
		(*b*) Describe any methods used to examine subgroups and interactions	Methods 4	Statistical analyses, multivariable logistic regression, confounding control, and diagnostics described.
		(*c*) Explain how missing data were addressed	NA	NA
		(*d*) *Cohort study*—If applicable, explain how loss to follow-up was addressed<br>*Case-control study*—If applicable, explain how matching of cases and controls was addressed<br>*Cross-sectional study*—If applicable, describe analytical methods taking account of sampling strategy	Methods 3	Sampling technique
		(*e*) Describe any sensitivity analyses	Methods 5	Statistical analyses, Multicollinearity<br>Model performance<br>goodness-of-fit test
**Results**				
Participants	13*	(a) Report numbers of individuals at each stage of study—eg numbers potentially eligible, examined for eligibility, confirmed eligible, included in the study,	Results	Participant flow and STROBE flow diagram (supp)

		completing follow-up, and analysed		
		(b) Give reasons for non-participation at each stage	Results	Participant flow and STROBE flow diagram (suppl)
		(c) Consider use of a flow diagram	NA	NA
Descriptive data	14*	(a) Give characteristics of study participants (eg demographic, clinical, social) and information on exposures and potential confounders	Results 5	Participant characteristics and descriptive statistics reported.
		(b) Indicate number of participants with missing data for each variable of interest	Results 5	Participant characteristics and descriptive statistics reported.
		(c) *Cohort study*—Summarise follow-up time (eg, average and total amount)	NA	NA
Outcome data	15*	*Cohort study*—Report numbers of outcome events or summary measures over time	NA	NA
		Case-control study—Report numbers in each exposure category, or summary measures of exposure	NA	NA
		Cross-sectional study—Report numbers of outcome events or summary measures	Results 5	Anemia prevalence and outcome data presented.
Main results	16	(*a*) Give unadjusted estimates and, if applicable, confounder-adjusted estimates and their precision (eg, 95% confidence interval). Make clear which confounders were adjusted for and why they were included	Results 5, 6	Unadjusted and adjusted odds ratios with 95% confidence intervals reported.
		(*b*) Report category boundaries when continuous variables were categorized	NA	NA
		(*c*) If relevant, consider translating estimates of relative risk into absolute risk for a meaningful time period	NA	NA

Other analyses	17	Report other analyses done—eg analyses of subgroups and interactions, and sensitivity analyses	NA	NA
**Discussion**				
Key results	18	Summarise key results with reference to study objectives	Discussion 7	Key findings summarized.
Limitations	19	Discuss limitations of the study, taking into account sources of potential bias or imprecision. Discuss both direction and magnitude of any potential bias	Discussion 8	Strengths and limitations discussed.
Interpretation	20	Give a cautious overall interpretation of results considering objectives, limitations, multiplicity of analyses, results from similar studies, and other	Discussion 7,8	Interpretation considers limitations and previous literature.

		relevant evidence		
Generalisability	21	Discuss the generalisability (external validity) of the study results	Discussion 8	Generalisability discussed.
**Other information**				
Funding	22	Give the source of funding and the role of the funders for the present study and, if applicable, for the original study on which the present article is based	Funding 8	Funding statement provided.

*Give information separately for cases and controls in case-control studies and, if applicable, for exposed and unexposed groups in cohort and cross-sectional studies.

Note: An Explanation and Elaboration article discusses each checklist item and gives methodological background and published examples of transparent reporting. The STROBE checklist is best used in conjunction with this article (freely available on the Web sites of PLoS Medicine at http://www.plosmedicine.org/, Annals of Internal Medicine at http://www.annals.org/, and Epidemiology at http://www.epidem.com/). Information on the STROBE Initiative is available at www.strobe-statement.org.
